# Volatiles Released by the Endophytic Fungus *Alternaria alstroemeriae* from *Vaccinium dunalianum* Promote the Growth of *Arabidopsis thaliana* and *Nicotiana benthamiana*

**DOI:** 10.3390/microorganisms14030639

**Published:** 2026-03-12

**Authors:** Yueyun Zhang, Wenhang Yin, Boyu Wu, Zhiyu Zhang, Guolei Zhu, Xiaoqin Yang, Fanrui Zhou, Imran Haider Shamsi, Ping Zhao, Lihua Zou

**Affiliations:** 1 National Joint Engineering Research Center for Highly-Efficient Utilization Technology of Forestry Resources, Southwest Forestry University, Kunming 650224, China; 2 Department of Food Science and Nutrition, College of Biosystems Engineering and Food Science, Zhejiang University, Hangzhou 310058, China; 3 Department of Agronomy, College of Agriculture and Biotechnology, Zhejiang University, Hangzhou 310058, China

**Keywords:** plant pathogenic fungi, *Alternaria* sp. strain, VOCs, plant growth promotion, omics technology

## Abstract

The study of volatile organic compounds (VOCs)-mediated plant growth promotion has long focused on various beneficial microbial species. As an important natural source of functional biomolecules, the biological function and potential value of VOCs released by plant pathogenic fungi in regulating plant growth still lack sufficient research, and further exploration is needed. In this study, a phytopathogenic fungus *Alternaria alstroemeriae* (strain Z84) was isolated from *Vaccinium dunalianum* for the first time, and the effects of its VOCs on the growth of *Arabidopsis thaliana* and *Nicotiana benthamiana* were systematically investigated. The results showed that after Z84 VOCs treatment, multiple phenotypic traits of the two plants were significantly improved, and the chlorophyll content was also markedly increased. Transcriptome analysis showed that a total of 1401 differentially expressed genes (DEGs) were identified in the treated *A. thaliana*, of which 629 were up-regulated and 772 were down-regulated. KEGG enrichment analysis showed that these DEGs were mainly enriched in photosynthesis-antenna proteins, plant–pathogen interaction, glutathione metabolism, plant hormone signal transduction, flavonoid biosynthesis and photosynthesis-related pathways. Metabolomics analysis revealed that Z84 VOCs treatment significantly changed the metabolic profile of *A. thaliana*, with the most significant changes in amino acid metabolism-related pathways. It is noteworthy that the plant hormone spectrum of *A. thaliana* was significantly changed after treatment, and the contents of salicylic acid (SA), abscisic acid (ABA) and gibberellins (GAs) were significantly up-regulated. These results not only demonstrate the potential of Z84-derived VOCs to facilitate plant growth but also provide an important basis for further dissecting the molecular mechanisms of plant–pathogenic fungi interactions.

## 1. Introduction

Global food security is a fundamental objective of the United Nations Sustainable Development Goals [1]. Escalating challenges arising from persistent population growth and the intensifying impacts of climate change increasingly threaten global food security. Concurrently, the overuse of chemical fertilizers and pesticides has led to pronounced soil degradation and environmental pollution, thereby driving an urgent need for sustainable agricultural practices [2]. Within this context, the development of environmentally friendly and sustainable green biofertilizers has become increasingly imperative [3]. In recent years, plant growth-promoting microorganisms (PGPMs), primarily comprising fungi and bacteria, have been shown to play an essential role in enhancing plant growth and development through multiple mechanisms, making them extremely valuable in sustainable agriculture [4].

PGPMs are key biological regulators in the plant microecosystem, encompassing genera such as *Bacillus* [5], *Pseudomonas* [6], and *Trichoderma* [7]. These beneficial microorganisms promote plant growth and development through diverse direct and indirect mechanisms, thereby enhancing plant phenotypic traits and stress tolerance. On the one hand, they can directly regulate plant cell division and elongation by secreting plant hormones [8] (such as auxins, gibberellins [9], and cytokinins), affecting the morphology of roots, stems, and leaves. On the other hand, they can indirectly optimize plant nutrient absorption and photosynthesis by enhancing nutrient uptake efficiency—such as dissolving insoluble phosphorus, synthesizing siderophores, assisting in nitrogen fixation, and providing trace elements like sulfur and iron [10]. For example, many essential nutrients that plants cannot directly utilize (such as nitrogen, phosphorus, potassium, and iron) can be converted by microorganisms into forms readily absorbable and utilizable, thus significantly promoting the acquisition and utilization efficiency of nutrients by plants [11,12]. In addition, some PGPMs can activate the plant’s antioxidant defense system [13], modulate secondary metabolic pathways such as phenylpropanoid metabolism [11], or inhibit pathogenic microorganisms by producing antibiotics [14], thereby systematically enhancing the host’s growth performance and disease resistance.

In addition, studies have shown that volatile organic compounds (VOCs) released by many microorganisms act as crucial signaling molecules in plant–microbe interactions that can significantly regulate plant growth, which has attracted widespread attention in the field of sustainable agriculture [15,16]. The growth-promoting properties of bacterial VOCs have been widely confirmed [17]. For instance, nitrogenous volatiles from *P. fluorescens* promote the growth of *Atractylodes lancea* [18]. Besides, the growth-promoting potential of fungal VOCs has been intensively studied. Their sources are extensive, encompassing not only beneficial fungal taxa such as *Trichoderma* sp. [19], *Papiliotrema flavescens* [20], and *Piriformospora indica* [21], but also pathogenic fungi. For instance, VOCs emitted by *T. atroviride* can up-regulate the expression levels of sucrose transporters and metabolic enzymes, thereby promoting the allocation of photoassimilates from shoots to roots and further significantly enhancing root growth in *A. thaliana* [22]. Similarly, VOCs produced by *P. flavescens* are capable of reshaping plant root system architecture via the auxin/ethylene signaling pathway and inducing systemic resistance through jasmonic acid/ethylene-mediated defense pathways; among these VOCs, acetoin, naphthalene, and indole are the key bioactive molecules mediating these physiological effects [23]. Additionally, VOCs emitted by the phytopathogenic fungus *Fusarium oxysporum* have been shown to significantly promote plant growth by disrupting the transport and signaling network of auxin within plants [24]. Similarly, volatiles released by the pathogenic fungus *Penicillium aurantiogriseum* can markedly alter root development by inducing a comprehensive reprogramming of the root proteome [25]. With advancing research, more efforts are expected to uncover a wider range of functional microbial resources [15], particularly regarding the growth-promoting effects of VOCs from pathogenic fungus, an area that remains significantly understudied.

The genus *Alternaria*, a group of widely distributed filamentous fungi [26], frequently acts as a plant pathogen in agricultural ecosystems [27,28], while simultaneously serving as valuable resources for bioactive metabolites [29]. In recent years, significant advances have been made in their metabolite’s characterization, biocontrol potential, and environmental adaptability. In the context of natural products development, perylenequinone derivatives, isolated from the marine sponge-derived fungus *Alternaria* sp. SCSIO41014, exhibit significant cytotoxicity towards three cancer cell lines and potent inhibitory activity against bacteria [30]. Chang et al. isolated against Epstein–Barr virus agents from the fungus *A. alstroemeriae* Km2286 [31]. Within the biocontrol realm, *Alternaria* sp. possess dual functions, with specific strains acting as biocontrol agents. For example, *A. alstroemeriae* YBG8, isolated from healthy *Lycium barbarum*, produces a fermentation filtrate that can significantly inhibit the growth of the root rot pathogen *F. solani* by destroying its cell wall and cell membrane integrity [32]. As a highly adaptable genus, *Alternaria* sp. thrive in stressful environments such as salinity stress conditions [33]. Although substantial progress has been made in the study of *Alternaria* sp., most of the work has focused on its non-volatile components, and it is still unclear whether its volatiles have growth-promoting effects on plants.

In this study, the effects of VOCs released by the endophytic fungus *A. alstroemeriae* Z84, which was isolated for the first time from *V. dunalianum*, on the growth of *A. thaliana* and *N. benthamiana* were systematically investigated using a divided Petri plate co-cultivation system. The underlying molecular mechanism of *A. alstroemeriae* Z84 VOCs promoting *A. thaliana* growth was elucidated by transcriptomics and metabolomics analysis. The results of this study laid a theoretical foundation for the application of *A. alstroemeriae* Z84 volatiles in sustainable agriculture and provided a scientific basis for exploring environmentally friendly agronomic measures to promote plant growth.

## 2. Materials and Methods

### 2.1. Isolation and Identification of Strain Z84

Strain Z84 was isolated from *V. dunalianum* collected in Wuding County, Chuxiong Yi Autonomous Prefecture, Yunnan Province, China. For molecular identification, genomic DNA of strain Z84 was extracted, and the ITS region was amplified with primers ITS1/ITS4 [34]. The amplification products were commercially sequenced (Tsingke Biotechnology, Beijing, China) and then subjected to BLASTn (BLAST: Basic Local Alignment Search Tool, https://blast.ncbi.nlm.nih.gov/Blast.cgi) analysis in the NCBI database. Phylogenetic tree was constructed using the maximum likelihood (ML) method in MEGA version 11.

### 2.2. Biological Materials and Cultivation Conditions

Wild-type *A. thaliana* (Col-0) and *N. benthamiana* seeds were kindly provided by the Kunming Institute of Botany, Chinese Academy of Sciences. Seed surface sterilization followed method of Hassani et al. [35] with slight modifications: Seeds were soaked in 75% (*v*/*v*) ethanol and shaken for 45 s, rinsed five times (45 s each time) with sterile distilled water, then soaked in 10% (*v*/*v*) sodium hypochlorite solution for 45 s, and finally rinsed five times with sterile distilled water (45 s each time). The sterilized seeds were evenly sown on a sterilized Petri plate containing 0.7% solidified Murashige and Skoog (MS) [36] medium (Basebio Biotechnology, Hangzhou, China) with 3.0% sucrose and vernalized for 2 days at 4 °C in the dark, and then were transferred to a controlled LED top-mounted artificial climate chamber (RDN-1000D, Ningbo Dongnan Instrument, Ningbo, China) for germination. The cultivation conditions were: temperature 22 °C, photoperiod 12 h light/12 h dark, and relative humidity 50–60%. The seedlings were used for further experiments after growing for 5 days.

Strain Z84 was cultured on potato sucrose agar (PSA) plates (containing 200 g/L potato, 18 g/L sucrose, and 10 g/L agar) and incubated at 28 °C for 7 days before being used in subsequent experiments.

### 2.3. Split Co-Culture Assay of Fungus and Plants

The fungal-plant co-culture experiments were conducted according to the method previously established [37]. The specific procedures were as follows: a divided Petri plate (90 mm × 16 mm; Bkmamlab, Changde, China) with a vertical central partition (3 mm thick, 10 mm high) was used for co-culture under sterile conditions. This central partition evenly divided the plate into two independent compartments of equal volume, physically isolating between the compartments while not affecting the diffusion of gas. Then 10 mL of PSA medium was added to side A of the divided Petri plate, and 10 mL of MS agar medium was added to side B. After the medium had completely solidified and cooled, five uniformly grown, 5-day-old *A. thaliana* seedlings were evenly transplanted onto the surface of the MS medium in side B. Using a 5 mm diameter punch, a piece of agar with mycelium was cut from the edge of a 7-day-old Z84 strain colony and inoculated into the center of the PSA medium in side A using an inoculation needle. Immediately after inoculation, the divided Petri plates were capped and sealed with two layers of Parafilm^®^ M sealing film (10 cm × 38 m) at the seam between the cap and the base. The divided Petri plates were then placed in the controlled LED top-mounted artificial climate chamber for incubation. The incubation conditions were set as follows: light intensity 10,000 LX, photoperiod 16 h light/8 h dark, incubation temperature 22 ± 1 °C, relative humidity 50–60%. All plates were incubated continuously for 14 days, with three biological replicates for each treatment. The divided Petri plate without strain Z84 inoculation served as a blank control. The co-culture experiments for *N. benthamiana* were performed under the same conditions as the *A. thaliana*, except that three seedlings were transplanted per plate on the B-side MS medium.

### 2.4. Pot Experiment

Seedlings of *A. thaliana* or *N. benthamiana* from the control group and the Z84 VOCs-treated group in the divided Petri plates co-culture system were transplanted into 5 cm × 5 cm pots. After culturing with strain Z84 in divided Petri plates for 14 days, uniformly growing *A. thaliana* or *N. benthamiana* seedlings from the control group and the Z84 VOCs-treated group were selected and transplanted into pots (5 cm × 5 cm). The cultivation substrate was a mixture of nutrient soil, vermiculite, and perlite in a 3:3:1 (*v*/*v*) ratio. One plant was planted per pot, with three biological replicates per group. After transplanting, the plants were placed in an artificial climate chamber for 14 days. The culture conditions were: light intensity of 10,000 LX, photoperiod of 16 h light/8 h darkness, temperature of 22 ± 1 °C, and relative humidity of 50–60%. After the culture period, the plants were harvested, and relevant growth parameters were measured.

### 2.5. Plant Growth Parameter Measurement

The seedlings of *A. thaliana* or *N. benthamiana* in the control group and Z84 VOCs-treated group were randomly selected for growth parameter measurement, and each group was set up with three independent biological replicates. The leaf area, primary root length and lateral root number were measured by ImageJ software (https://imagej.net/ij/). The determination method of plant fresh weight and dry weight referred to a previous study [38].

### 2.6. Analysis of Chlorophyll Content

Chlorophyll content was determined using the method of Zeb et al. [39] with slight modifications. Fresh leaves (0.2 g) from *A. thaliana* or *N. benthamiana* plants cultured in divided Petri plates (14 days) and pots (14 days), respectively, were homogenized and extracted with 80% acetone in a 25 mL volumetric flask. The extracts were kept in darkness until the leaf tissue turned completely white. Three independent biological replicates were performed for each treatment. The absorbance of the extracts was then measured at 663 nm, 646 nm, and 470 nm. Chlorophyll concentrations were calculated using the following formulas:

Chlorophyll a = 12.21A_663_ − 2.81A_646_

Chlorophyll b = 20.13A_646_ − 5.03A_663_

Total chlorophyll = Chlorophyll a + Chlorophyll b

### 2.7. Determination of VOCs

Headspace solid-phase microextraction combined with gas chromatography–mass spectrometry (HS-SPME-GC-MS) was employed to examine the VOCs emitted by strain Z84. An extraction head composed of divinylbenzene/carboxen/polydimethylsiloxane StableFlex was used for compound collection, following a specific extraction protocol: initially, the samples were placed in a water bath at 22 °C for 20 min, then a 3 mm-diameter hole was drilled in the Petri dish using a small electric drill, and the SPME head (purchased from Merck KGaA, Darmstadt, Germany) was inserted into this hole for a 40 min extraction. Afterwards, the extracted compounds were desorbed in the injector port of the GC-MS instrument (Agilent, Santa Clara, CA, USA) at 250 °C for 5 min. GC-MS analysis was performed according to the method described in previous study [38].

### 2.8. Transcriptomic Sequencing Analysis

The shoots of *A. thaliana* seedlings that were cocultured with or without strain Z84 VOCs for 14 days were selected as transcriptome sequencing materials, and three independent biological replicates were set for each group.

The total RNA was extracted by using the Trizol reagent kit (Invitrogen, Carlsbad, CA, USA), and the RNA concentration was detected by Nanodrop 2000 spectrophotometer (Thermo Fisher Scientific, Waltham, MA, USA). RNA integrity was evaluated by RNase-free agarose gel electrophoresis and the Agilent 2100 bioanalyzer (Agilent Technologies, Palo Alto, CA, USA). Oligo (dT) magnetic beads were used to enrich fungal mRNA. After being interrupted by fragmentation buffer, the cDNA library was synthesized by reverse transcription using NEBNext Ultra RNA library preparation kit (NEB # 7530, New England Biolabs, Ipswich, MA, USA). The resulting cDNA libraries were sequenced on the Illumina NovaSeq 6000 platform by Genedenovo Biotechnology Co., Ltd. (Guangzhou, China).

### 2.9. Metabolites Identification and Quantification

The samples used for metabolomics analysis were identical to those used for transcriptome. Three independent biological replicates were performed per group. The freeze-dried samples were homogenized using a mixer mill (MM 400, Retsch, Haan, Germany) with zirconia beads at 30 Hz for 1.5 min. Subsequently, 100 mg of the resulting powder was accurately weighed and extracted with 1.0 mL of 70% methanol aqueous solution containing 0.1 mg/L lidocaine as internal standard at 4 °C overnight. After centrifugation at 10,000× *g* for 10 min, the supernatants were filtered through 0.22 μm filter membrane prior to ultra-high performance liquid chromatography–tandem mass spectrometry (UPLC-MS/MS) analysis.

Metabolite analysis was performed using LC-MS/MS. The instrumentation system consisted of a shimadzu SHIM-pack UFLC CBM30A UPLC unit (Shimadzu, Kyoto, Japan) coupled with an Applied Biosystems 6500 QTRAP mass spectrometer (Applied Biosystems, Waltham, MA, USA). Chromatographic separation was achieved using a Waters ACQUITY UPLC HSS T3 C18 column (2.1 × 100 mm, 1.8 μm) (Waters, Milford, MA, USA) at a column temperature of 40 °C and a flow rate of 0.4 mL/min. The mobile phase consisted of 0.04% aqueous acetic acid (A) and acetonitrile (B), with the following gradient elution program: 0 min, 95% A; 11.0 min, 5% A; 12.0 min, 5% A; 12.1 min, 95% A; 15.0 min, 95% A.

Mass spectrometric detection was carried out using an ESI-triple quadrupole-linear ion trap (QTRAP) mass spectrometer (AB Sciex QTRAP6500, SCIEX, Framingham, MA, USA) equipped with an ESI-Turbo Ion-Spray interface operating in positive ion mode. Instrument control and data acquisition were performed using Analyst 1.6.1 software (AB Sciex). The key MS parameters were set as follows: ESI source temperature, 500 °C; ion spray voltage, 5500 V; curtain gas, 25 psi; collision-activated dissociation (CAD), high. QQQ scans were conducted in multiple reaction monitoring (MRM) mode with individually optimized declustering potential (DP) and collision energy (CE) for each transition. The mass scan range was set to *m/z* 50–1000.

Raw metabolic data were processed via Progenesis QI software v3.1 for peak extraction, alignment, normalization and metabolite annotation against public databases (HMDB, KEGG). Orthogonal partial least squares-discriminant analysis (OPLS-DA) was conducted to obtain the variable importance in projection (VIP) values, and DAMs were screened with the integrated criteria of VIP ≥ 1, |log_2_ fold change (FC)| ≥ 1 and *p* < 0.05 (Student’s *t*-test).

### 2.10. Statistical Analysis

Data are presented as the mean ± standard deviation of three independent biological replicates. One-way ANOVA was performed using SPSS 23.0 software for comparisons between the Z84 VOCs-treated and untreated groups, and Duncan’s multiple range test was used to correct for multiple comparisons to determine the significance of differences between groups. *p* < 0.05 was considered statistically significant.

## 3. Results

### 3.1. VOCs Produced by Strain Z84 Promoted the Growth of A. thaliana and N. benthamiana Seedlings

**Figure 1:**
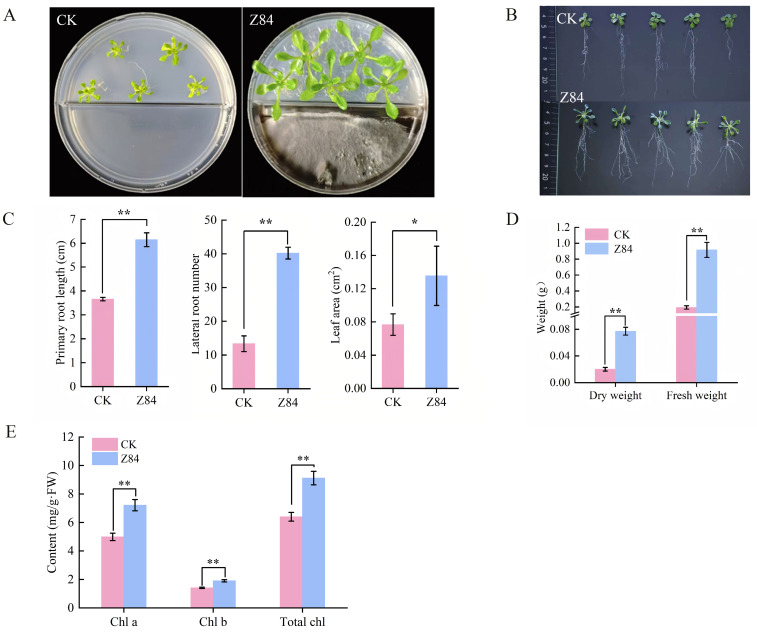
Effects of VOCs from strain Z84 on growth indices of *A. thaliana* seedlings. (**A**) Overall growth. (**B**) Root architecture. (**C**) Root parameters and leaf area. (**D**) Dry weight and fresh weight. (**E**) Chlorophyll content. CK: control group (without Z84 inoculation); Z84: treatment group (with Z84 inoculation). Chl a: chlorophyll a; Chl b: chlorophyll b; Total Chl: total chlorophyll. Data are presented as the mean ± standard deviation (SD) of three biological replicates (*n* = 3). Asterisks indicate the significance level of difference according to Duncan’s multiple range test (* *p* < 0.05; ** *p* < 0.01).

To investigate the effects of VOCs produced by strain Z84 on plant growth, a non-contact co-culture system using a split Petri plate was employed to co-culture plant seedlings exposed to Z84-derived VOCs, These seedlings exhibited significantly enhanced overall growth compared with the control (Figure 1A). As shown in Figure 1B, Z84 VOCs treatment significantly promoted the root architecture of *A. thaliana*. Compared with the control, the primary root length, lateral root number, and leaf area of the treated *A. thaliana* were increased by 67.93%, 200%, and 72.17%, respectively (Figure 1C). Accordingly, significant increases in shoot biomass were observed, with dry weight and fresh weight increasing by 282.95% and 374.19%, respectively, relative to the control (Figure 1D). The content of chlorophyll a, chlorophyll b, and total chlorophyll of the treated *A. thaliana* were increased by 44.59%, 35%, and 42.48%, respectively (Figure 1E) compared with the control. The increased chlorophyll content implied an enhanced capacity for light energy absorption and utilization.

**Figure 2:**
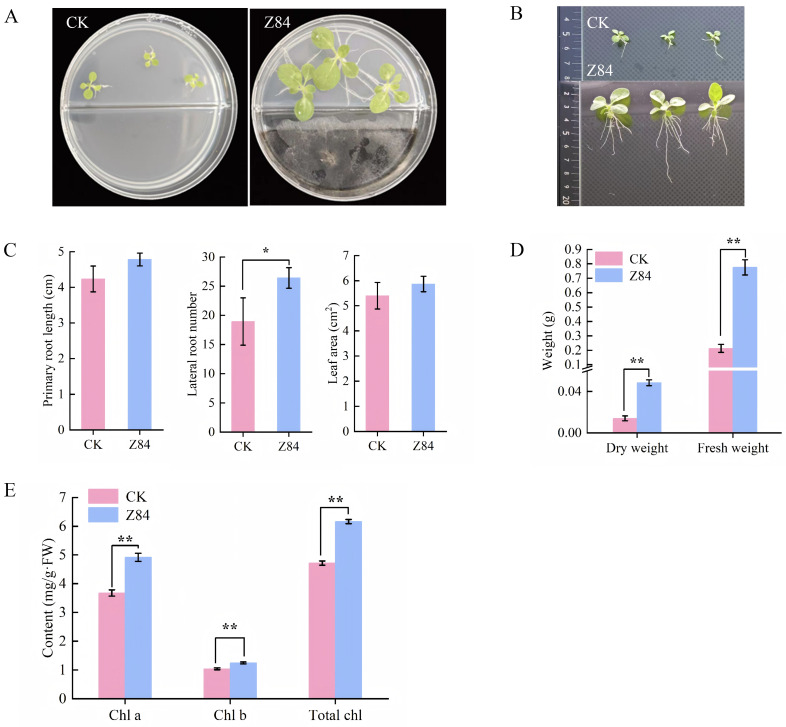
The effects of VOCs from strain Z84 on the growth indices of *N. benthamiana* seedlings. (**A**) Overall growth. (**B**) Root architecture. (**C**) Primary root length and lateral root number and leaf area. (**D**) Dry weight and fresh weight. (**E**) Chlorophyll content. CK: control group (without Z84 inoculation); Z84: treatment group (with Z84 inoculation). Chl a: chlorophyll a; Chl b: chlorophyll b; Total Chl: total chlorophyll. Data are presented as the mean ± standard deviation (SD) of three biological replicates (*n* = 3). Asterisks indicate the significance level of difference according to Duncan’s multiple range test (* *p* < 0.05; ** *p* < 0.01).

Similarly, the VOCs produced by Z84 also significantly promote the growth of *N. benthamiana*. As shown in Figure 2A, *N. benthamiana* seedlings exposed to Z84-produced VOCs displayed significantly superior overall growth compared with the control. Root system architecture analysis demonstrated that the Z84 VOCs-treated *N. benthamiana* plants significantly outperformed the control in primary root length, lateral root number, and shoot biomass (Figure 2B). Specifically, primary root length increased by 13%, lateral root number by 39.46%, and leaf area by 10.37% (Figure 2C). Shoot biomass was also significantly increased, with dry weight and fresh weight elevated by 245.95% and 265.24%, respectively (Figure 2D). Additionally, Z84 VOCs treatment significantly increased chlorophyll content in *N. benthamiana* leaves, with the contents of chlorophyll a, chlorophyll b, and total chlorophyll elevated by 33.63%, 19.72%, and 30.56%, respectively, compared with the control (Figure 2E).

### 3.2. The VOCs Produced by Strain Z84 Showed a Sustainable Growth-Promoting Effect on A. thaliana and N. benthamiana

**Figure 3:**
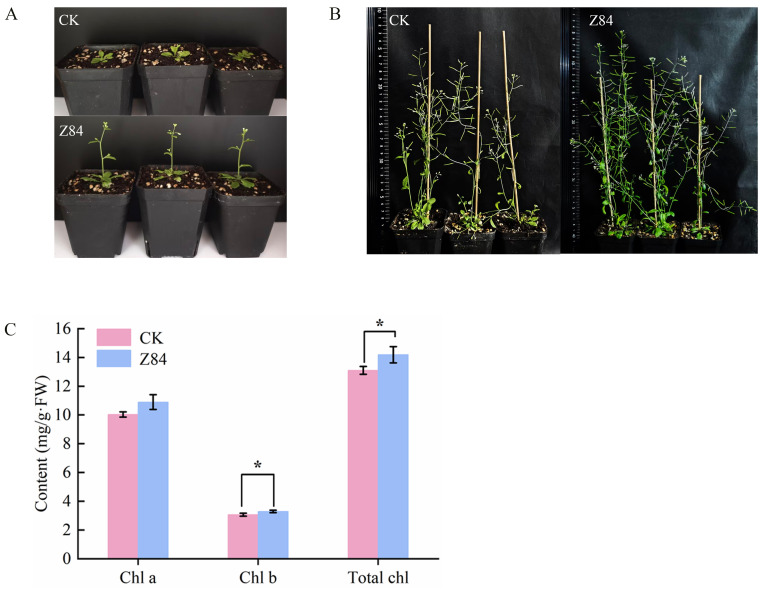
The growth changes in *A. thaliana* after removing Z84 VOCs. (**A**) Overall growth and flowering period. (**B**) Silique and seed setting number. (**C**) Chlorophyll content. CK: control group (without Z84 VOCs exposure); Z84: treatment group (with Z84 VOCs removed after exposure). Chl a: chlorophyll a; Chl b: chlorophyll b; Total Chl: total chlorophyll. Data are presented as the mean ± standard deviation (SD) of three biological replicates (*n* = 3). Asterisks indicate the significance level of difference according to Duncan’s multiple range test (* *p* < 0.05).

To evaluate the sustainability of the growth-promoting effects induced by VOCs from strain Z84, the subsequent growth of *A. thaliana* plants was monitored after removal of VOCs from Z84. Phenotypic analysis showed that even after 7 days of removal of Z84 VOCs, the treatment group still maintained a significant growth advantage and flowered earlier than the control group (Figure 3A). After 30 days of transplantation culture, the overall growth of the *A. thaliana* in the treatment group continued to be better than that of the control (Figure 3B), with higher biomass accumulation. It is worth noting that the treatment group also exhibited more siliques and a higher seed setting rate (Figure 3B). Even after removal of Z84 VOCs, the chlorophyll content in the *A. thaliana* leaves of the treatment group was still significantly higher than that of the control group (Figure 3C). The VOCs produced by Z84 not only promoted the growth of *A. thaliana* during co-culture, but also continued to play an important role after removal, showing a significant sustainable effect.

**Figure 4:**
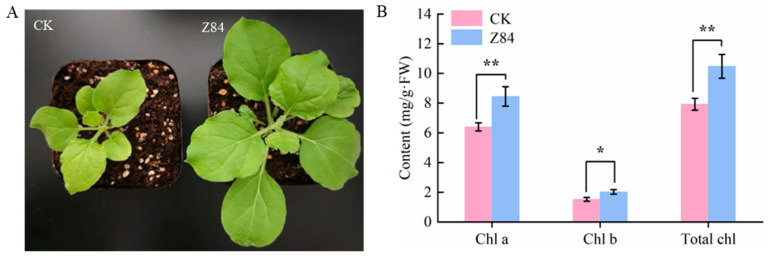
The growth changes in *N. benthamiana* after removing Z84 VOCs. (**A**) Overall growth. (**B**) Chlorophyll content. CK: control group (without Z84 VOCs exposure); Z84: treatment group (with Z84 VOCs removed after exposure). Chl a: chlorophyll a; Chl b: chlorophyll b; Total Chl: total chlorophyll. Data are presented as the mean ± standard deviation (SD) of three biological replicates (*n* = 3). Asterisks indicate the significance level of difference according to Duncan’s multiple range test (* *p* < 0.05; ** *p* < 0.01).

A sustained growth-promoting effect of VOCs from strain Z84 was also observed in the *N. benthamiana*. After removal of VOCs from Z84, *N. benthamiana* in the treated group still exhibited significantly superior overall growth compared to the control, specifically manifested as expanded leaf area and increased leaf number (Figure 4A). The chlorophyll level in the leaves of the *N. benthamiana*-treated group was significantly higher than that in the control (Figure 4B), indicating that the functional activity of the photosynthetic system was maintained at a higher level even after the removal of Z84 VOCs. These results indicated that the VOCs released by strain Z84 also had a sustained growth-promoting effect on *N. benthamiana* plants. The VOCs produced by Z84 likely achieve this sustained, cross-species growth-promoting effect by activating endogenous physiological regulatory pathways in plants, even after the direct exposure phase has ended. These findings provide important support for its potential application in agriculture.

### 3.3. Identification of Strain Z84 VOCs

Although this study has observed significant growth-promoting effects of strain Z84 VOCs on *A. thaliana* and *N. benthamiana* seedlings, the specific composition of these VOCs remains to be clarified. Therefore, this study employed HS-SPME-GC-MS to further identify and analyze of the VOCs produced by strain Z84, and the detailed information of the detected volatiles is shown in Table 1. The results indicated that terpenoids were the main components of strain Z84 VOCs, with (−)-thujopsene and (+)-beta-cedrene as the major components, accounting for 84.65% and 15.35% of the total relative content, respectively.

### 3.4. Morphological Characteristics and Molecular Identification of Strain Z84

**Figure 5:**
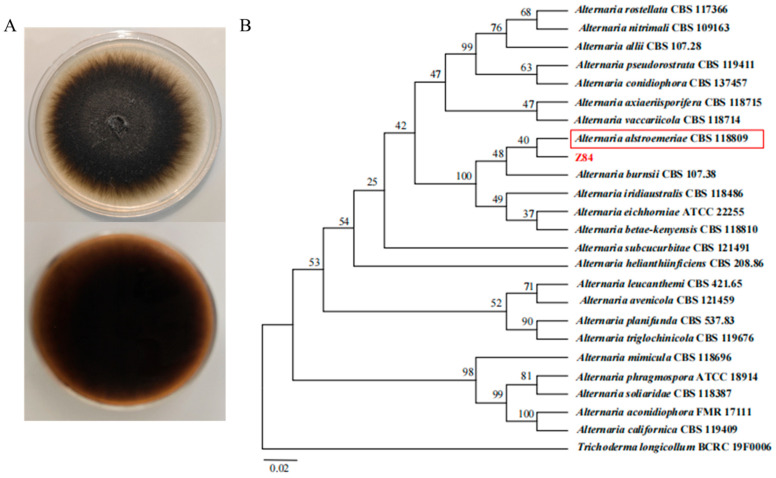
Morphological and phylogenetic analysis of strain Z84, (**top**): front side; (**bottom**): back side. (**A**) Colony morphology of strain Z84 cultured on PSA medium. (**B**) Phylogenetic tree of strain Z84 and other fungal strains constructed based on ITS sequence analysis. The red box indicates strains clustered in the same clade as strain Z84. The scale bar represents a genetic distance of 0.02.

The colony of strain Z84 on PSA medium is round, initially grayish-white to olive-colored. As the incubation time increases, the number of spores gradually increases, and the colony color progressively deepens to dark olive-brown, eventually approaching black. The mycelium exhibits a cottony or velvety, with a uniform texture, while the reverse side of the colony usually exhibits a characteristic dark brown to black color (Figure 5A). The morphological features of the present isolate were similar to a previously published description [30] of *A. alstroemeriae*.

Molecular identification based on the ITS rDNA sequence revealed that strain Z84 shared the highest sequence similarity with other isolates of the genus *Alternaria*. Phylogenetic analysis further demonstrated that Z84 formed a distinct clade with *Alternaria alstroemeriae* strain CBS118809, which clearly separated it from other taxonomic groups (Figure 5B). Integrating the morphological characteristics with molecular phylogenetic evidence, the strain was conclusively identified as *A. alstroemeriae*.

### 3.5. Transcriptome Analysis of A. thaliana in Response to Z84 VOCs

**Figure 6:**
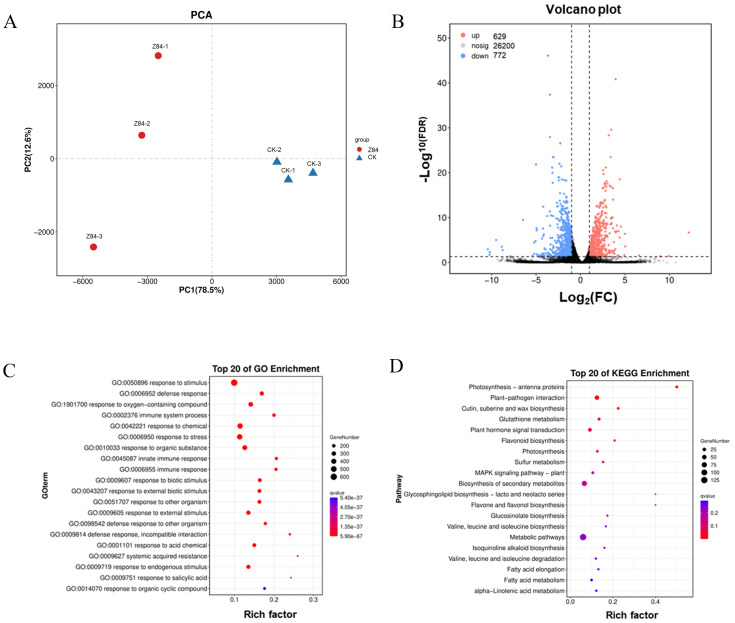
Transcriptome analysis of *A. thaliana* in response to strain Z84 VOCs. (**A**) Principal component analysis (PCA). (**B**) DEGs volcano plot. (**C**) GO enrichment analysis of DEGs. (**D**) KEGG pathway enrichment analysis of DEGs.

Although the above results show that the VOCs produced by strain Z84 can promote the growth of *A. thaliana* and *N. benthamiana*, the specific growth-promoting mechanism is still unclear. To further analyze the growth-promoting mechanism of VOCs, the model plant *A. thaliana* with a well-annotated genome was selected for transcriptome sequencing analysis. The results of principal component analysis (PCA) showed that the control group and the Z84 VOCs-treated group exhibited distinct separation in gene expression profiles (Figure 6A), indicating that Z84 VOCs treatment significantly changed the transcriptome characteristics of *A. thaliana*. Based on the established thresholds (|log_2_(Fold Change)| > 1 and FDR < 0.05), a total of 1401 differentially expressed genes (DEGs) were identified, including 629 up-regulated genes and 772 down-regulated genes (Figure 6B).

Gene Ontology (GO) enrichment analysis of the DEGs (Figure 6C) showed that biological processes related to stimulus response, defense response, immune response, stress response, and systemic acquired resistance were significantly enriched. The top 20 KEGG enrichment analysis (Figure 6D) indicated that the DEGs were significantly enriched in pathways including photosynthesis-antenna proteins, plant–pathogen interaction, glutathione metabolism, plant hormone signal transduction, flavonoid biosynthesis, photosynthesis, sulfur metabolism, MAPK signaling pathway-plant and glucosinolate biosynthesis (Figure 6D). Among these, glutathione metabolism, flavonoid biosynthesis, and plant hormone signal transduction pathways are closely related to plant stress resistance, suggesting that these pathways play a synergistic role in coordinating plant growth and stress adaptation.

### 3.6. Analysis of Genes Regulated by Z84 VOCs in A. thaliana

**Figure 7:**
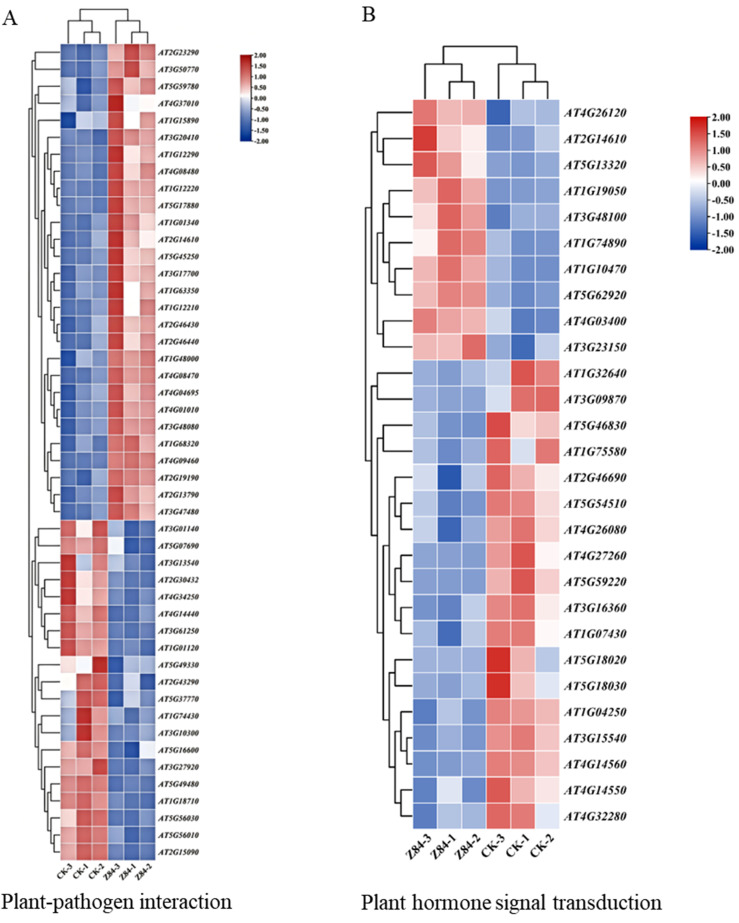

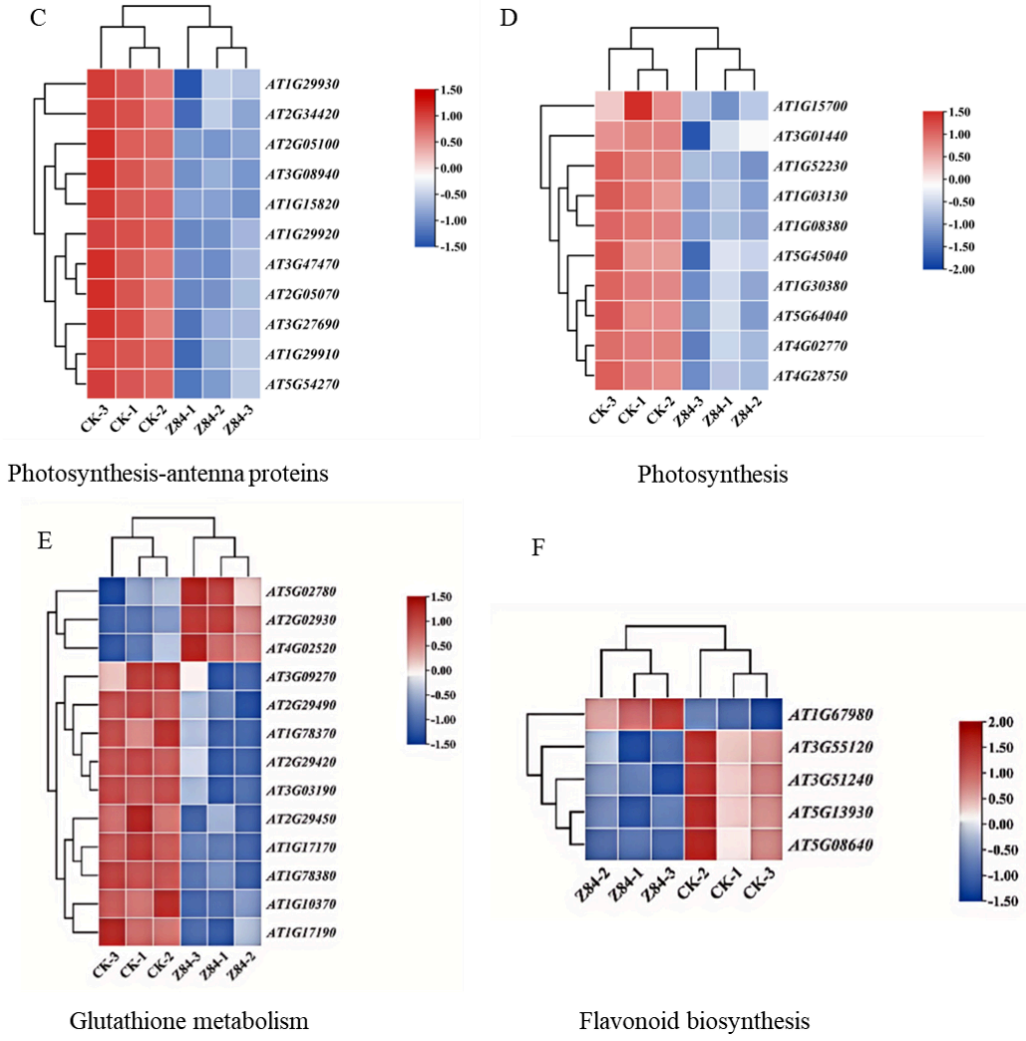
Heatmap of gene expression levels in six major metabolic pathways. (**A**) Plant–pathogen interaction. (**B**) Plant hormone signal transduction. (**C**) Photosynthesis-antenna proteins. (**D**) Photosynthesis. (**E)** Glutathione metabolism. (**F**) Flavonoid biosynthesis.

The expression pattern analysis of DEGs within the enriched KEGG pathways showed that the plant–pathogen interaction and plant hormone signal transduction pathways contained the largest number of DEGs. In the plant–pathogen interaction pathway (Figure 7A, Table S1), most genes were significantly up-regulated. Among them, the *CML* genes encoding calmodulin-like proteins, a plant-specific protein family, including *CML41* and *CML47*, showed the most significant up-regulation, with the expression levels of *CML41* increased by 7.37-fold and *CML47* by 6.06-fold, respectively. Additionally, the expression of the conserved R2R3-type MYB transcription factor *MYB62* in plants was up-regulated by 6.59-fold, while that of alpha/beta-hydrolases superfamily protein EDS1B was elevated by 4.75-fold. In contrast, *TCL1*, an R3 MYB transcriptional repressor, and *GL1*, an R2R3 MYB transcriptional activator, displayed notably down-regulated expression, with their expression levels reduced by 23.43-fold and 4.69-fold, respectively. In the plant hormone signal transduction pathway (Figure 7B, Table S1), compared with the control group, Z84 VOCs treatment significantly up-regulated several key genes: *ARR6*, *ARR7*, and *ARR15*—which encode type-A response regulators—by 7.72-fold, 4.69-fold, and 4.29-fold, respectively; *GH3.12*, encoding an acyl acid amido synthetase belonging to subgroup III of the *A. thaliana GH3* family, was elevated by 4.76-fold; and the gene *At2g14610* encoding pathogenesis-related protein 1 (PR-1) was up-regulated by 4.19-fold. The up-regulated expression of these genes indicated that Z84 VOCs treatment could effectively activate the plant immune responses against pathogens.

In addition, all genes involved in the photosynthesis-antenna proteins pathway were significantly down-regulated, including those encoding the core components of the photosystem II (PSII) light-harvesting complex II (LHCII). For instance, the expression levels of *LHCB2.4*, *LHCB1.1*, and *LHCB2.2* were reduced by 13.38-fold, 12.67-fold, and 8.21-fold, respectively. This gene expression pattern clearly reflects the growth–defense trade-off strategy adopted by plants in response to Z84 VOCs treatment. Furthermore, genes associated with photosynthesis (Figure 7C, Table S1) were also significantly down-regulated. For example, genes (*PSAD2*, *PSAD1*, and *ATPC2*) encoding the photosystem I (PSI) D subunit showed a global down-regulation trend, suggesting that Z84 VOCs treatment exerted a significant inhibitory effect on photosynthesis in *A. thaliana*. Besides, the expression levels of most genes in the glutathione metabolic pathway were decreased under Z84-derived VOCs treatment, of which *GSTU24* was the most significantly down-regulated, by 8.70-fold (Figure 7E, Table S1). In the flavonoid biosynthesis pathway, the majority of genes exhibited down-regulated expression patterns. Among them, *At1g6798*, which encodes caffeoyl-CoA O-methyltransferase, and a core enzyme gene *FLS1* in the flavonoid biosynthesis pathway were most significantly down-regulated, and their expression levels were reduced by 5.80-fold and 4.98-fold, respectively (Figure 7F, Table S1). These results demonstrated that Z84 VOCs could markedly activate the plant–pathogen interaction and plant hormone signal transduction pathways in *A. thaliana*, and induce the up-regulated expression of disease resistance-related genes, thereby enhancing the immune defense capacity of the plants. Meanwhile, to balance the defense requirements, the plants down-regulate the expression of genes associated with photosynthesis-antenna proteins, photosynthesis, and flavonoid biosynthesis, thus exhibiting a typical growth–defense trade-off strategy.

### 3.7. Effects of Z84 VOCs Treatment on Endogenous Plant Hormones in A. thaliana

**Figure 8:**
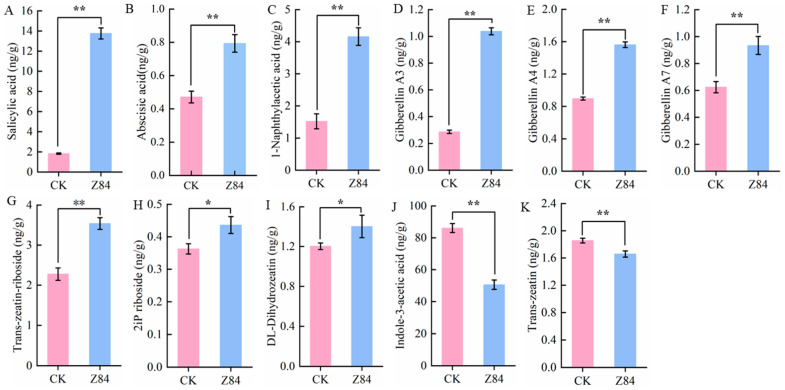
Content of plant hormones with significant changes in *A. thaliana* before and after strain Z84 VOCs treatment. (**A**) Salicylic acid. (**B**) Abscisic acid. (**C**) 1-Naphthylacetic acid. (**D**) Gibberellin A3. (**E**) Gibberellin A4. (**F**) Gibberellin A7. (**G**) Trans-zeatin-riboside. (**H**) 2iP riboside. (**I**) DL-Dihydrozeatin. (**J**) Indole-3-acetic acid. (**K**) Trans-zeatin. CK: control group (without inoculation of strain Z84); Z84: treatment group (with inoculation of strain Z84). Data are presented as the mean ± standard deviation (SD) of three biological replicates (*n* = 3). Asterisks indicate the significance level of difference according to Duncan’s multiple range test (* *p* < 0.05; ** *p* < 0.01).

Given the high abundance and heterogeneous expression of DEGs in the plant hormone signal transduction pathway, the contents of phytohormones in the aboveground tissues of *A. thaliana* of the treatment and control groups were determined. These results indicated that Z84 VOCs treatment led to the up-regulation of multiple phytohormone contents in *A. thaliana*: specifically, salicylic acid (SA) was increased by 7.59-fold (Figure 8A, Table 2), abscisic acid (ABA) by 1.68-fold (Figure 8B, Table 2), 1-naphthaleneacetic acid (1-NAA) by 2.75-fold (Figure 8C, Table 2), gibberellin A_3_ (GA_3_) by 3.62-fold (Figure 8D, Table 2), gibberellin A_4_ (GA_4_) by 1.74-fold (Figure 8E, Table 2), and gibberellin A_7_ (GA_7_) by 1.50-fold (Figure 8F, Table 2). Additionally, the contents of trans-zeatin riboside (Figure 8G, Table 2), isopentenyladenosine (2iP riboside) (Figure 8H, Table 2) and DL-dihydrozeatin (Figure 8I, Table 2) were all increased by 1.0-fold, all of which showed significantly higher levels than those in the control group. Among these, SA exhibited the most prominent increase in content. However, the contents of indole-3-acetic acid (IAA) (Figure 8J, Table 2) and trans-zeatin (Figure 8K, Table 2) were significantly lower than those in the control group (Figure 8J,K, Table 2), with their levels down-regulated by 1.7-fold and 1.22-fold, respectively.

These results indicated that Z84 VOCs treatment could remarkably remodel the hormone homeostasis of *A. thaliana*, which was specifically reflected by its differential regulatory effects on different phytohormones. On the one hand, this treatment significantly induced the accumulation of most phytohormones including SA and gibberellins (GA_3_/GA_4_/GA_7_), among which SA exhibited the most prominent up-regulation, implying its core regulatory role in the physiological processes mediated by Z84 VOCs. On the other hand, the treatment significantly inhibited the biosynthesis and accumulation of indole-3-acetic acid (IAA) and trans-zeatin, which reflected the synergistic regulatory features of the plant hormone signal transduction pathway. Combined with the previously observed differential expression patterns of genes involved in the plant hormone signal transduction pathway, these findings further confirmed that Z84 VOCs could mediate the trade-off between plant growth, development, and immune defense by regulating hormone metabolism and signal transduction.

### 3.8. Z84 VOCs Induced Metabolome Reprogramming in A. thaliana

**Figure 9:**
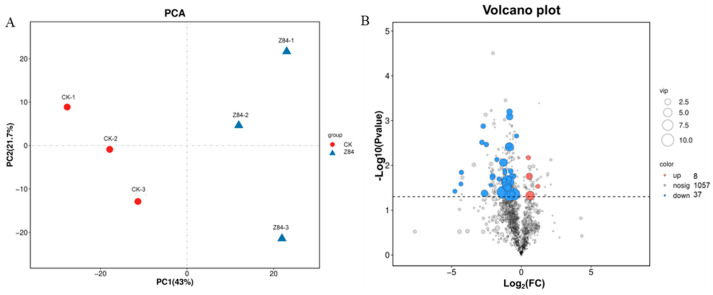

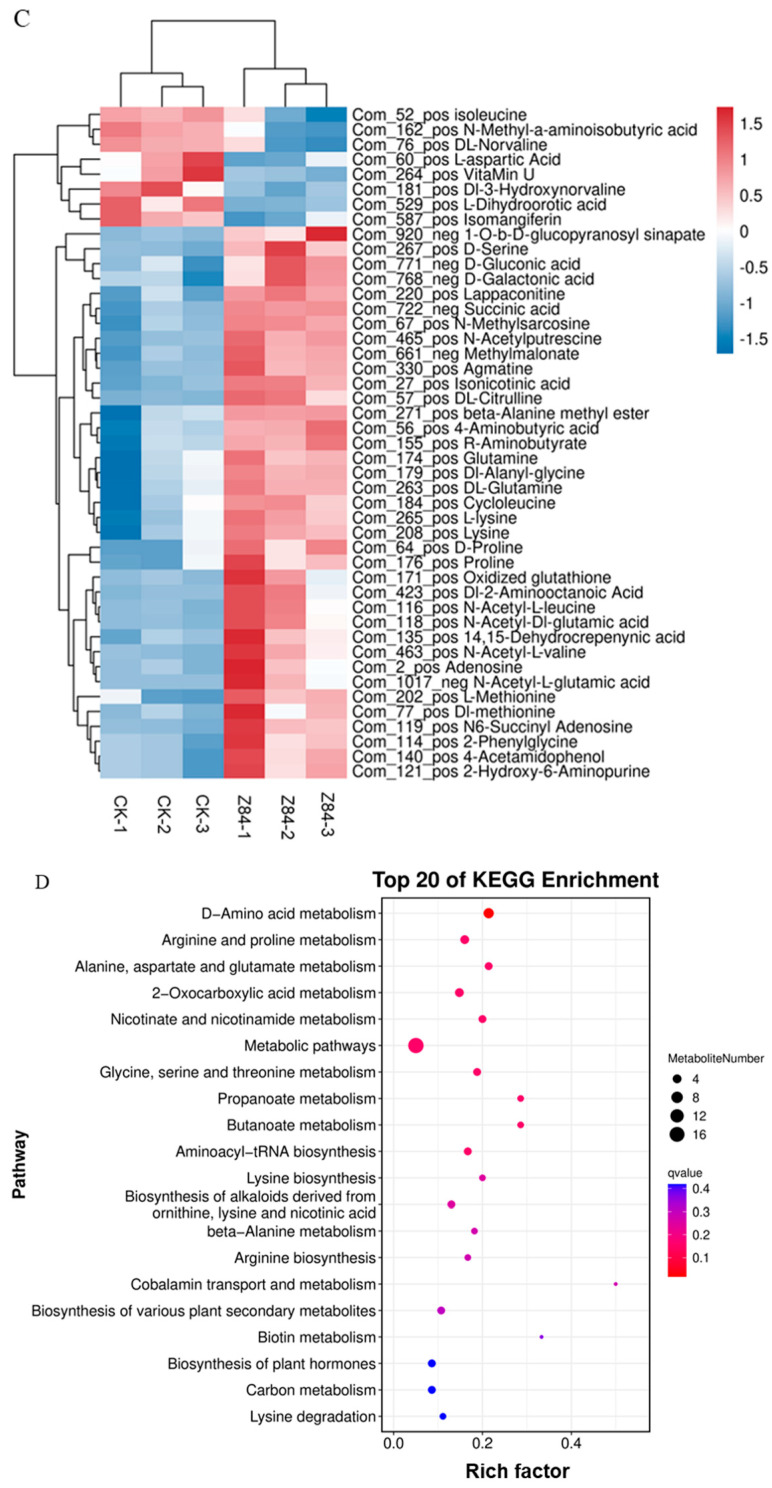
Metabolomic profiling of *A. thaliana* in response to Z84 VOCs. (**A**) Principal component analysis (PCA). (**B**) DEMs volcano plot. (**C**) Heatmap of DEM expression patterns. (**D**) KEGG pathway enrichment analysis of DEMs.

LC-MS/MS-based metabolome analysis was conducted to comprehensively characterize the metabolome reprogramming induced by Z84 VOCs treatment in *A. thaliana*. PCA results indicated a significant difference in metabolomic profiles between the Z84 VOCs-treated and control groups (Figure 9A). A total of 45 differentially expressed metabolites (DEMs) were identified, among which eight metabolites were significantly up-regulated and 37 metabolites were significantly down-regulated (Figure 9B). The heatmap visually displayed the differential expression patterns of individual DEMs (Figure 9C).

KEGG pathway enrichment analysis revealed that DEMs were significantly enriched in pathways such as D-amino acid metabolism, arginine and proline metabolism, alanine, aspartate and glutamate metabolism, 2-oxocarboxylic acid metabolism, and nicotinate and nicotinamide metabolism (Figure 9D). Specifically, the D-amino acid metabolic pathway contained six differential metabolites. Among them, five metabolites (L-lysine, L-methionine, N-acetyl-L-glutamic acid, D-serine, and D-proline) were significantly down-regulated, while only L-aspartic acid was significantly up-regulated (Table 3). In the arginine and proline metabolism pathway, agmatine, 4-aminobutyric acid, 5-aminobutyric acid, and 6-aminobutyric acid all exhibited significant down-regulation. In the alanine, aspartate and glutamate metabolism pathway, succinic acid and 4-aminobutyric acid were significantly down-regulated, whereas L-aspartic acid was significantly up-regulated. Collectively, these results indicated that Z84 VOCs treatment significantly reshaped the metabolome of *A. thaliana*, with core changes concentrated on amino acid metabolism-related pathways.

## 4. Discussion

The use of microbial VOCs as eco-friendly bioinoculants has emerged as a promising strategy for sustainable agriculture, given their ability to regulate plant growth and stress resistance without direct contact with plants [40]. In this study, *A. alstroemeriae* strain Z84 was identified as a novel VOCs-producing fungus that significantly promotes the growth of *A. thaliana* and *N. benthamiana* seedlings. The morphological and molecular characterization of Z84 is consistent with the taxonomic features of *Alternaria* sp., which are widely distributed diverse ecosystems worldwide, and this fungal genus known for its role as a plant pathogen [41]. Notably, most previous studies on *Alternaria* sp. have focused on their pathogenicity [40] and the bioactivity of non-volatile metabolites [42,43]; these findings reveal previously unrecognized growth-promoting potential of *A. alstroemeriae* VOCs, expanding the known functional diversity of this fungal genus.

The growth-promoting effects of Z84 VOCs were manifested in multiple phenotypic traits, including enhanced root architecture, increased leaf area, and elevated biomass accumulation. Specifically, Z84 VOCs increased the primary root length of *A. thaliana* by 67.93% and enhanced fresh weight by 374.19%. This growth-promoting effect was comparable to or even more pronounced than that induced by VOCs from well-characterized beneficial fungi. For instance, volatiles emitted by *T. asperelloides* strain PSU-P1 have been demonstrated to significantly promote the growth of *A. thaliana*, increasing its fresh weight by 28.5% and root length by 23.08% [19]. A key contributing factor to this growth advantage is the elevated chlorophyll content. Chlorophyll content is critical for light energy absorption and photosynthetic efficiency [44]. Notably, *A. thaliana* plants treated with VOCs from the pathogenic fungus *A. alternata* showed similar chlorophyll accumulation [45]. Transcriptome analysis revealed enrichment of photosynthesis-related pathways, suggesting that Z84 VOCs may regulate the photosynthetic system to boost carbon fixation and energy supply for plant growth [46]. However, most DEGs in photosynthesis-antenna proteins and photosynthesis pathways were down-regulated.

This apparent contradiction can be explained by negative feedback regulation and growth–defense trade-off optimization. Excess chlorophyll may lead to photooxidative damage, so plants down-regulate photosynthesis-related genes to avoid redundant synthesis of light-harvesting proteins, while sufficient chlorophyll maintains high photosynthetic efficiency [47,48]. Meanwhile, transcriptome and hormone analyses showed activated plant–pathogen interaction and hormone signaling pathways, with strongly increased SA and GA_3_ levels. To balance defense and growth, plants reallocate resources from redundant photosynthesis gene expression to biomass production and defense responses, consistent with the increased plant biomass and up-regulated growth/defense hormones [49,50].

Separately, a striking finding of this study was the sustainability of the growth-promoting effect of Z84 VOCs. Even after the removal of Z84 VOCs, *A. thaliana* and *N. benthamiana* plants maintained significant growth advantages. This sustainability distinguishes Z84 VOCs from transient growth stimulants, and suggests that they induce long-term physiological reprogramming in plants. Similar persistent effects have been reported for VOCs from *Piriformospora indica* [21]. It was thought that the underlying mechanism of this phenomenon is that after sensing external stimuli, plants initiate a plant defense response, which in turn triggers coordinated changes at multiple levels, including physiology, transcription, metabolism, and epigenetics. This activation effect can typically be maintained for a long time and spans the entire life cycle of the plant, thus exhibiting a lasting impact [51]. These transcriptome data support this hypothesis, as Z84 VOCs induced extensive transcriptional reprogramming involving 1401 DEGs, including pathways related to metabolism, hormone signaling, and stress response. These DEGs may serve as “memory” markers that the growth-promoting phenotype, even after the initial VOC stimulus, was removed. The sustained growth-promoting effect of Z84 strain VOCs on plants suggests its potential for practical application in field environments. Future research should focus on field trials to systematically evaluate the stability and persistence of this growth-promoting effect under natural soil conditions, providing crucial theoretical support for its agricultural application.

Terpenes were identified as the dominant VOCs emitted by strain Z84, with (+)-beta-cedrene and (−)-thujopsene as the core components. A previous study has demonstrated that cedrene, a sesquiterpene produced by beneficial fungi *T. guizhouense*, stimulates *A. thaliana* root development through auxin signaling and transport [52]. Furthermore, another major terpenoid component discovered in this study—(−)-thujopsene—has also been shown to participate in regulating root architecture and nutrient uptake efficiency [53]. Terpenes, as a crucial class of microbial volatiles, have been widely recognized as key regulators of plant growth and stress responses [54]. Thus, terpenes identified in this study are likely the core functional substances responsible for its plant growth-promoting effects. Further in-depth functional verification and mechanism analysis of these two compounds are needed to provide a theoretical basis for their development and application in sustainable agriculture.

Transcriptome analysis revealed significant enrichment of DEGs in plant–pathogen interaction and plant hormone signal transduction, indicating that Z84 VOCs modulate plant hormone-related metabolism to promote growth. Plant hormones play a central role in mediating growth-promoting effects of microbial VOCs [55]. These results showed that Z84 VOCs significantly altered the endogenous hormone profile of *A. thaliana*, with most hormones showing a significant up-regulation trend. The up-regulation of salicylic acid (SA) was particularly significant. SA has been widely reported for its important function in systemically induced resistance (ISR) [56], and a recent study has found that moderate concentrations of SA can promote plant growth [57]. The simultaneous up-regulation of SA and other growth-related hormones (e.g., GA3, GA4, zeatin riboside) suggested that Z84 VOCs can synergistically regulate plant growth and defense responses, demonstrating an optimized growth–defense trade-off [58].

It is noteworthy that while Z84 VOCs promoted plant growth, they were accompanied by a significant decrease in IAA (down-regulated by 1.7-fold) and trans-zeatin (down-regulated by 1.22-fold) levels, seemingly contradicting their traditional understanding as classic growth hormones. However, this apparent inconsistency is resolved by the fine-tuned hormone crosstalk and resource reallocation within the context of the growth–defense trade-off. First, the down-regulation of IAA and trans-zeatin is compensated by the up-regulation of other key hormones: GAs (GA_3_: up-regulated by 3.62-fold, GA_4_: up-regulated) directly promote cell elongation and division, while cytokinin derivatives (trans-zeatin riboside, isopentenyladenosine) substitute for trans-zeatin in regulating meristem activity [59], collectively maintaining the growth momentum. Secondly, the moderate down-regulation of IAA enhances lateral root development by reducing apical dominance [60], which is consistent with the 200% increase in lateral root number of *A. thaliana*, indicating that Z84 VOCs fine-tune IAA levels to optimize root architecture rather than simply promoting IAA accumulation. In addition, the down-regulation of IAA and trans-zeatin reduces the metabolic cost of excessive growth signal transduction, allowing more resources to be redirected to defense-related processes (e.g., SA biosynthesis, PR protein expression) and core growth processes (e.g., GA-mediated cell expansion), thus balancing growth and defense demands [61]. For example, GAs are key regulators of cell elongation and division [62], and their up-regulation likely contributes to the increased root length and leaf area observed in Z84-treated plants. In contrast, IAA was down-regulated, which may seem counterintuitive given its role in root growth. However, recent studies have shown that moderate down-regulation of IAA can enhance lateral root development by reducing apical dominance [63], which is consistent with the observed increase in lateral root number of *A. thaliana*. This suggested that Z84 VOCs fine-tune the balance of multiple hormones to optimize root architecture and overall plant growth.

The enrichment of DEGs in the plant–pathogen interaction pathway and the up-regulation of SA imply that Z84 VOCs may also enhance plant resistance to pathogens in addition to promoting growth. The down-regulation of calcium signaling-related genes (*CML41*, *CML47*) and stress-responsive genes (*NIG1*, *HAI1*) in the plant–pathogen interaction and plant hormone signal transduction pathways further supported the idea that Z84 VOCs create a favorable growth environment by reducing plant stress responses, allowing more resources to be allocated to growth [64]. This dual function of promoting growth and enhancing resistance makes Z84 VOCs highly valuable for the development of integrated green pest management and high-efficiency production systems.

Metabolome analysis identified 45 differential metabolites, with most amino acids (e.g., proline, glutamine, lysine) significantly down-regulated in the Z84-treated group. Amino acids are not only building blocks for protein synthesis but also serve as signaling molecules and precursors for secondary metabolites [65]. The down-regulation of most amino acids may reflect their efficient utilization for biomass accumulation (e.g., protein synthesis, cell wall formation) rather than passive accumulation [66], which is consistent with the increased plant biomass observed in the treatment group. Notably, oxidized glutathione, a key metabolite involved in plant redox homeostasis, was significantly down-regulated. This suggested that Z84 VOCs reduce oxidative stress in plants, as oxidized glutathione accumulates under stress conditions, and its reduction indicates improved redox balance [67]. This was further supported by the down-regulated expression of glutathione S-transferase genes *GSTF11* and *GSTU24* in the glutathione metabolism pathway, because GSTs are primarily involved in detoxification and stress response [68].

## 5. Conclusions

VOCs released by a phytopathogenic fungus Z84, firstly isolated from *V. dunalianum*, can significantly promote the growth of *A. thaliana* and *N. benthamiana*. The growth-promoting effect was sustained even after the removal of Z84 VOCs. Morphological and molecular characterization indicated that strain Z84 belongs to *A. alstroemeriae*. Transcriptomics analysis showed that the growth-promoting effect of Z84 VOCs on *A. thaliana* was closely associated with the regulation of key physiological processes such as plant–pathogen interaction, plant hormone signal transduction, and photosynthesis. Metabolomics analysis demonstrated significant alterations in amino acid metabolism and endogenous phytohormone levels. SA was significantly up-regulated, and the accumulation of gibberellins (GA3, GA4, and GA7) was increased in treated *A. thaliana*. These findings provide a new perspective for dissecting the molecular mechanisms underlying plant–phytopathogenic fungus interactions, and offer microbial resource and theoretical support for further exploration of plant growth regulation strategies in sustainable agriculture.

## Figures and Tables

**Table 1:** The VOCs emitted from strain Z84 were identified by using SPME-GC-MS.

Compound	PubChem ID	Molecular Formula	Molecular Weight	Relative Content (%)
(+)-beta-cedrene	11106485	C_15_H_24_	204.35	15.35 ± 1.45
(−)-thujopsene	442402	C_15_H_24_	204.35	84.65 ± 1.45

**Table 2:** Comparison of phytohormone levels in *A. thaliana* shoots between strain Z84 VOCs-treated and untreated groups.

Compound	RT (min)	CK-Mean (ng·g^−1^ FW)	Z84-Mean (ng·g^−1^ FW)	Log2_FC	*p*
Indole-3-acetic acid	1.1	86.12	50.59	−0.77	0.0001
Salicylic acid	4.88	1.83	13.76	2.91	3.0850
1-Naphthylacetic acid	6.11	1.52	4.16	1.45	0.0002
Trans-zeatin-riboside	2.71	2.28	3.54	0.64	0.0005
Gibberellin A3	3.98	0.29	1.04	1.86	1.5311
Gibberellin A4	6.25	0.90	1.56	0.80	8.4520
Abscisic acid	5.12	0.47	0.79	0.75	0.0009
Gibberellin A7	6.16	0.62	0.93	0.58	0.0024
Trans-zeatin	1.65	1.85	1.66	−0.16	0.0040
DL-Dihydrozeatin	1.74	1.20	1.40	0.22	0.0432
2iP riboside	4.02	0.36	0.44	0.27	0.0139

**Table 3:** Significantly enriched metabolic pathways of DEMs after Z84 VOCs treatment.

	Pathway ID	Pathway Name	Number of DEMs	Metabolites	Log2_FC
1	ko00470	D-Amino acid metabolism	6	L-Lysine	−1.03
				L-Aspartic acid	0.67
				L-Methionine	−1.60
				N-Acetyl-L-glutamic acid	−4.76
				D-Serine	−1.75
				D-Proline	−0.98
2	ko00330	Arginine and proline metabolism	4	Agmatine	−2.82
				4-Aminobutyric acid	−0.76
				D-Proline	−0.98
				N-Acetylputrescine	−2.73
3	ko00250	Alanine, aspartate and glutamate metabolism	3	Succinic acid	−0.83
				L-Aspartic acid	0.67
				4-Aminobutyric acid	−0.76
4	ko01210	2-Oxocarboxylic acid metabolism	4	L-lysine	−1.03
				L-Aspartic acid	0.67
				L-Methionine	−1.60
				N-Acetyl-L-glutamic acid	−4.76
5	ko00760	Nicotinate and nicotinamide metabolism	3	Succinic acid	−0.83
				L-Aspartic acid	0.67
				4-Aminobutyric acid	−0.76
6	ko01100	Metabolic pathways	17	Succinic acid	−0.83
				L-Lysine	−1.03
				L-aspartic acid	0.67
				L-Methionine	−1.60
				Oxidized glutathione	−2.64
				Agmatine	−2.82
				Adenosine	−0.53
				D-Gluconic acid	−0.90
				4-Aminobutyric acid	−0.76
				L-Dihydroorotic acid	0.52
				N-Acetyl-L-glutamic acid	−4.76
				D-Serine	−1.75
				D-Proline	−0.98
				D-Galactonic acid	−0.87
				N-Methylsarcosine	−0.33
				Methylmalonate	−0.93
				N-Acetylputrescine	−2.73
7	ko00260	Glycine, serine and threonine metabolism	3	L-Aspartic acid	0.68
				D-Serine	−1.75
				N-Methylsarcosine	−0.33
8	ko00640	Propanoate metabolism	2	Succinic acid	−0.83
				Methylmalonate	−0.93
9	ko00650	Butanoate metabolism	2	Succinic acid	−0.83
				4-Aminobutyric acid	−0.76
10	ko00970	Aminoacyl-tRNA biosynthesis	3	L-Lysine	−1.03
				L-Aspartic acid	0.67
				L-Methionine	−1.60

## Data Availability

The raw sequencing data of this study has been submitted to the National Genome Data Center (NGDC) with accession number CRA038670.

## Supplementary Materials

The following supporting information can be downloaded at: https://www.mdpi.com/article/10.3390/microorganisms14030639/s1, Table S1: Significantly enriched KEGG pathways of DEGs in shoots of Z84 VOCs-treated *A. thaliana* seedlings compared with untreated controls.
