# Supplementary material for: Volatiles Released by the Endophytic Fungus Alternaria alstroemeriae from Vaccinium dunalianum Promote the Growth of Arabidopsis thaliana and Nicotiana benthamiana
Source: Microorganisms. 2026 Mar 12;14(3):639. doi: 10.3390/microorganisms14030639 (PMC13029499; doi:10.3390/microorganisms14030639)
Supplement: Supplementary file 1 [file microorganisms-14-00639-s001.zip › microorganisms-4145851-supplementary.pdf]

Table1 S1. Significantly enriched KEGG pathways of DEGs in shoots of Z84 VOCs-treated *A. thaliana* seedlings compared with untreated controls.

Pathway	Symbol	Description	log2(fc)
Plant-pathogen interaction	CML41	calmodulin-like 41	2.87
	CML47	calcium-binding EF-hand family protein	2.73
	MYB62	myb domain protein 62	2.69
	EDS1B	alpha/beta-Hydrolases superfamily protein	2.24
	SIRK	FLG22-induced receptor-like kinase 1	2.12
	CNGC10	cyclic nucleotide gated channel 10	2.12
	At2g14610	pathogenesis-related gene 1	2.07
	CNGC11	cyclic nucleotide-gated channels	1.91
	CML19	centrin 2	1.70
	MEKK3	MAPK/ERK kinase kinase 3	1.67
	CNGC3	cyclic nucleotide gated channel 3	1.63
	MYB73	myb domain protein 70	1.46
	At1g12290	disease resistance protein (CC-NBS-LRR class) family	1.40
	MYB59	myb domain protein 59	1.28
	CNGC13	cyclic nucleotide-gated channel 13	1.28
	CNGC20	cyclic nucleotide-binding transporter 1	1.21
	RPS5	disease resistance protein (CC-NBS-LRR class) family	1.15
	MYB2	myb domain protein 112	1.14
	SERK4	somatic embryogenesis receptor-like kinase 4	1.12

CPK31	calcium-dependent protein kinase 31	1.11
RFL1	RPS5-like 1	1.10
At1g15890	disease resistance protein (CC-NBS-LRR class) family	1.09
RPS4	disease resistance protein (TIR-NBS-LRR class) family	1.08
At1g63350	disease resistance protein (CC-NBS-LRR class) family	1.06
MEKK2	mitogen-activated protein kinase kinase kinase 9	1.05
CPK9	calmodulin-domain protein kinase 9	1.044
CSA1	disease resistance protein (TIR-NBS-LRR class)	1.02
MYB6	myb domain protein 6	1.01
CML5	calcium-binding EF-hand family protein	-1.03
MYB111	myb domain protein 111	-1.09
KCS16	3-ketoacyl-CoA synthase 16	-1.10
CML24	EF hand calcium-binding protein family	-1.10
MYB20	myb domain protein 43	-1.11
CML49	calcium-binding EF-hand family protein	-1.12
MYB17	myb domain protein 17	-1.12
MYB106	myb domain protein 95	-1.13
KCS1	3-ketoacyl-CoA synthase 1	-1.15
HSP90-2	heat shock protein 81-2	-1.27
MYB106	myb domain protein 106	-1.28
KCS8	3-ketoacyl-CoA synthase 8	-1.32
ECI3	3-hydroxyacyl-CoA dehydratase 1	-1.57

	HSP90-3	heat shock protein 81-3	-1.59
	CP1	Ca2+-binding protein 1	-1.61
	MYB5	myb domain protein 5	-1.62
	MYB29	myb domain protein 29	-1.10
	MYB106	myb domain protein 47	-2.17
	GL1	myb domain protein 0	-2.40
	TCL1	homeodomain-like superfamily protein	-4.55
Plant hormone signal transduction	ARR6	response regulator 6	2.93
	GH3.12	auxin-responsive GH3 family protein	2.25
	ARR7	response regulator 7	2.23
	ARR15	response regulator 15	2.10
	At2g14610	pathogenesis-related gene 1	2.07
	ARR5	response regulator 5	2.00
	NPR2	ankyrin repeat family protein / BTB/POZ domain-containing protein	1.52
	ETR2	signal transduction histidine kinase, hybrid-type, ethylene sensor	1.33
	GH3.10	auxin-responsive GH3 family protein	1.26
	ARR4	response regulator 4	1.16
	SAUR32	SAUR-like auxin-responsive protein family	-1.01
	ABI1	protein phosphatase 2C family protein	-1.09

	IAA14	indole-3-acetic acid inducible 14	-1.15
	GH3.6	auxin-responsive GH3 family protein	-1.17
	SAUR50	SAUR-like auxin-responsive protein family	-1.19
	GH3.5	auxin-responsive GH3 family protein	-1.37
	IAA1	indole-3-acetic acid inducible	-1.48
	SAUR32	SAUR-like auxin-responsive protein family	-1.49
	IAA19	indole-3-acetic acid inducible 19	-1.64
	IAA29	indole-3-acetic acid inducible 29	-1.64
	IAA17	AUX/IAA transcriptional regulator family protein	-1.67
	MYC2	basic helix-loop-helix (bHLH) DNA-binding family protein	-1.94
	SAUR20	SAUR-like auxin-responsive protein family	-2.11
	AIP1	highly ABA-induced PP2C gene 2	-2.32
	AHP4	HPT phosphotransmitter 4	-2.67
	SAUR21	SAUR-like auxin-responsive protein family	-2.73
	SAG113	highly ABA-induced PP2C gene 1	-3.16
	BHLH28	NACL-inducible gene 1	-3.92
Photosynthesis - antenna proteins	LHCB1.3	chlorophyll A/B binding protein 1	-1.33
	LHCA4	light-harvesting chlorophyll-protein complex I subunit A4	-1.44
	LHCB4.2	light harvesting complex photosystem II	-1.48
	CAP10B	light harvesting complex photosystem II subunit 6	-2.16

Photosynthesis	LHCB1.3	photosystem II light harvesting complex gene B1B2	-2.30
	LHCB2.1	photosystem II light harvesting complex gene 2.1	-2.37
	LHCB3	light-harvesting chlorophyll B-binding protein 3	-2.41
	LHCB2.2	photosystem II light harvesting complex gene 2.2	-3.03
	LHCB1.1	chlorophyll A/B binding protein 3	-3.46
	LHCB1.1	chlorophyll A/B-binding protein 2	-3.67
	LHCB2.4	photosystem II light harvesting complex gene 2.3	-3.74
	ATPC2	ATPase, F1 complex, gamma subunit protein	-1.64
	PSAD2	photosystem I subunit D-2	-1.37
	psaD1	photosystem I subunit D-1	-1.148
	PSAE1	photosystem I reaction centre subunit IV / PsaE protein	-1.15
	PSAH2	photosystem I subunit H2	-1.44
	PSAK	photosystem I subunit K	-1.52
	PSAN	photosystem I reaction center subunit PSI-N, chloroplast, putative / PSI-N, putative (PSAN)	-1.10
	PNSL3	PsbQ-like 1	-1.06
	PETJ	cytochrome c	-1.03
	PSAO	photosystem I subunit O	-1.49
Glutathione metabolism	GSTU17	glutathione S-transferase family protein	-2.14
	GSTU24	glutathione S-transferase TAU 24	-3.12
	GSTU26	glutathione S-transferase tau 26	-1.36
	GSTU20	glutathione S-transferase TAU 20	-2.56

	GSTU19	glutathione S-transferase TAU 19	-1.10
	GSTF3	glutathione S-transferase F3	2.32
	GSTU7	glutathione S-transferase tau 7	-1.70
	GSTU5	glutathione S-transferase tau 5	-1.33
	GSTU1	glutathione S-transferase TAU 1	-2.54
	GSTF11	glutathione S-transferase F11	-3.43
	GSTU8	glutathione S-transferase TAU 8	-1.70
	GSTF2	glutathione S-transferase PHI 2	1.48
	GSTL1	glutathione transferase lambda 1	1.28
Flavonoid biosynthesis	F3H	flavanone 3-hydroxylase	-1.66
	At1g67980	caffeoyl-CoA 3-O-methyltransferase	2.53
	CHS	chalcone and stilbene synthase family protein	-1.94
	CHI1	chalcone-flavanone isomerase family protein	-1.04
	FLS1	flavonol synthase 1	-2.34
